# Supplementary material for: A spatial multi-omics atlas of immunosenescence reveals germinal-center B cell dysfunction in human lymph nodes
Source: Cell Press Blue. Author manuscript; Available in PMC 2026 Jul 21. (PMC13384480; doi:10.1016/j.cpblue.2026.100053)
Supplement: 1 [file NIHMS2194313-supplement-1.pdf]

CPBLUE, Volume *1*

Supplemental information

A spatial multi-omics atlas of immunosenescence reveals germinal-center B cell dysfunction in human lymph nodes

Negin Farzad, Archibald Enninful, Yao Lu, Fabio Parisi, Anthony Fung, Yumi Kwon, Yajuan Li, Margaux Labrosse, Mingyu Yang, Francesco Strino, Liang Chen, Junchen Yang, Mei Zhong, Fu Gao, Bo Tao, Joseph Cunningham, Zhiliang Bai, Haikuo Li, Fang Wang, Michael Stankewich, Dongjoo Kim, Mingze Dong, Lisa M. Bramer, Keyi Li, Meera R. Bhat, Evan Loe, Joseph Craft, Ljiljana Pasa-Tolic, Stephanie Halene, Lingyan Shi, Yuval Kluger, Mina L. Xu, and Rong Fan

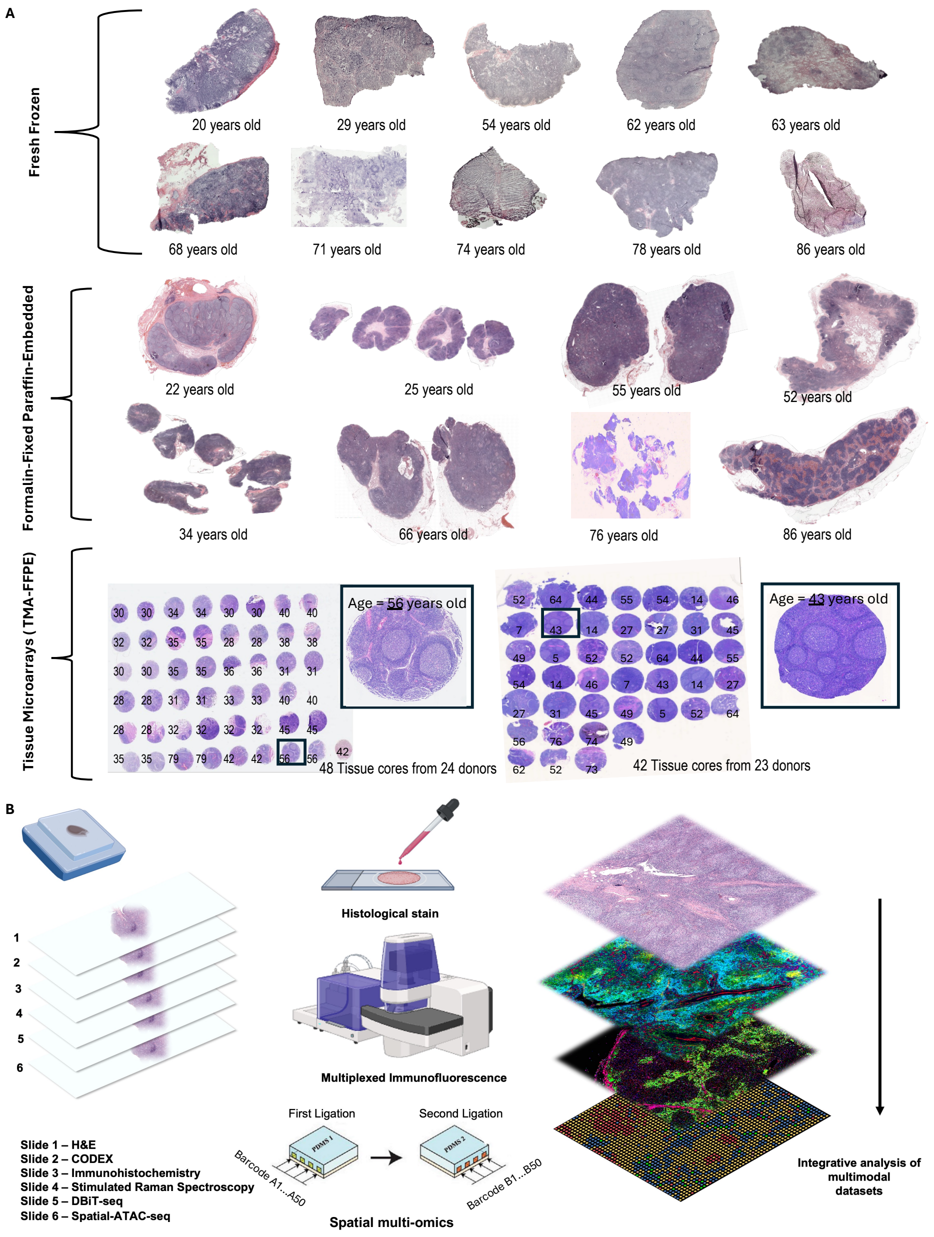

Figure S1. Overview of human lymph node samples and multimodal spatial profiling workflow. (A) Representative hematoxylin and eosin (H&E)-stained images of human lymph node sections collected from donors of varying ages, displayed by preservation method. Fresh frozen samples (top) include donors aged 20–86 years. Formalin-fixed paraffin-embedded (FFPE) samples (middle) include donors aged 22–86 years. Tissue microarrays (TMAs, bottom) consist of multiple cores from 47 donors (ages indicated), with representative enlarged cores. (B) Schematic of the integrative multimodal spatial profiling workflow. Serial tissue sections were processed for histological staining (Slide 1: H&E), multiplexed immunofluorescence imaging (Slide 2: CODEX), immunohistochemistry (Slide 3), stimulated Raman spectroscopy (Slide 4), DBiT-seq (Slide 5), and spatial-ATAC-seq (Slide 6). Spatial barcoding and ligation enable multi-omics data integration for comprehensive analysis of cellular and molecular features within human lymph node tissues.

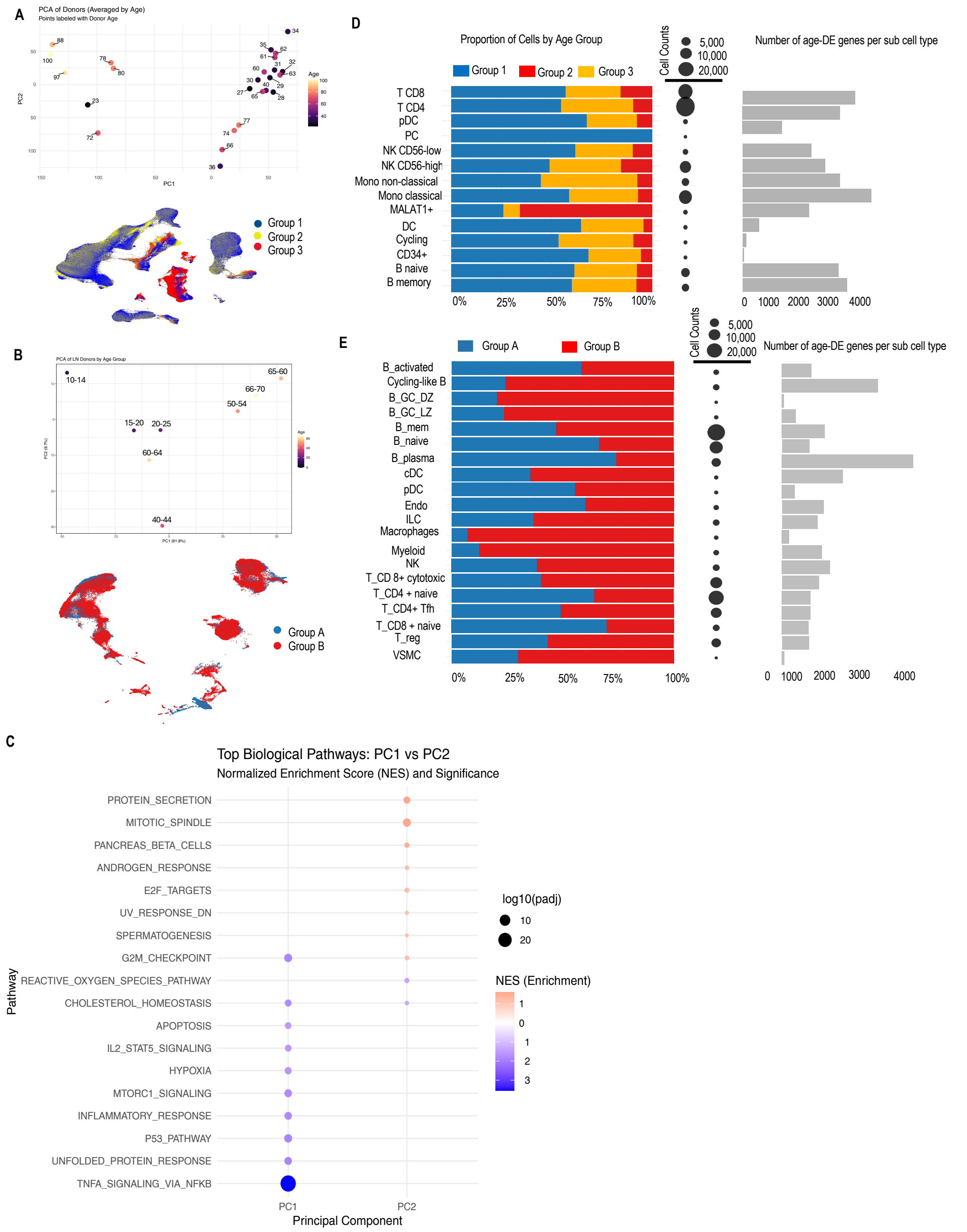

Figure S2. Single cell-RNA-seq data set with district sub cell types and across different age group

(A,B) Principal component analysis (PCA) of PBMC and lymphoid (SLO) donors across ages shows segregation along PC1 and PC2 , with age-associated separation across samples. UMAP projections colored by age group (Group A and B in lymphoid , Group 1, Group 2, Group 3 in pbmc) illustrate the distribution of cells in each group.

(C) Dot plot showing top biological pathways enriched along PC1 and PC2 based on normalized enrichment score (NES). Dot size represents statistical significance (log10 adjusted p-value), and color indicates enrichment direction and magnitude.

(D&E)Stacked bar plots display the proportion of cell types for each dataset across age groups (Group 1–3). Dot size indicates total cell counts per cell type, and horizontal bar plots show the number of age-associated differentially expressed genes (DEGs) per sub-cell type.

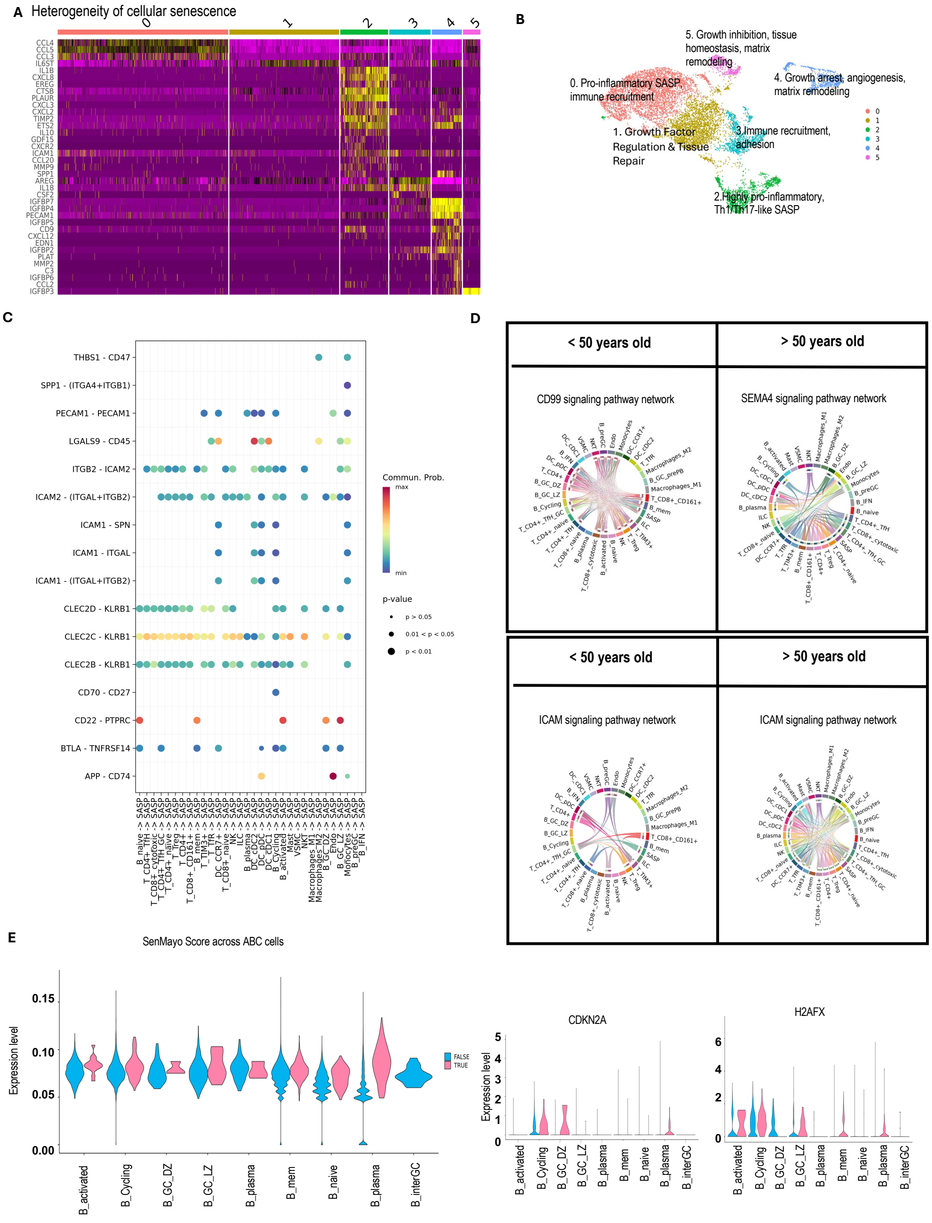

Figure S3. Cellular heterogeneity, ligand-receptor interactions, and age-associated changes in senescent B cell networks.

(A) Heatmap showing heterogeneity of cellular senescence across clusters, highlighting differential expression of senescence-associated genes in distinct immune cell populations.

(B) UMAP plot depicting six major senescent cell clusters annotated by their dominant functional signatures.

(C) Dot plot showing significant ligand–receptor interactions between SASP cells and other immune cell types in donors aged more than 50 years. The color scale represents communication probability, and dot size corresponds to p-value significance.

(D) Chord diagrams comparing age-associated differences in signaling pathway networks (<50 vs. >50 years old), illustrating enrichment of CD99, SEMA4, and ICAM signaling pathways in each group.

(E) Violin plots displaying SenMayo module scores and expression levels of CDKN2A (p16) and H2AFX across age-associated B cell subsets, showing increased senescence signatures in specific B cell populations in aged individuals.

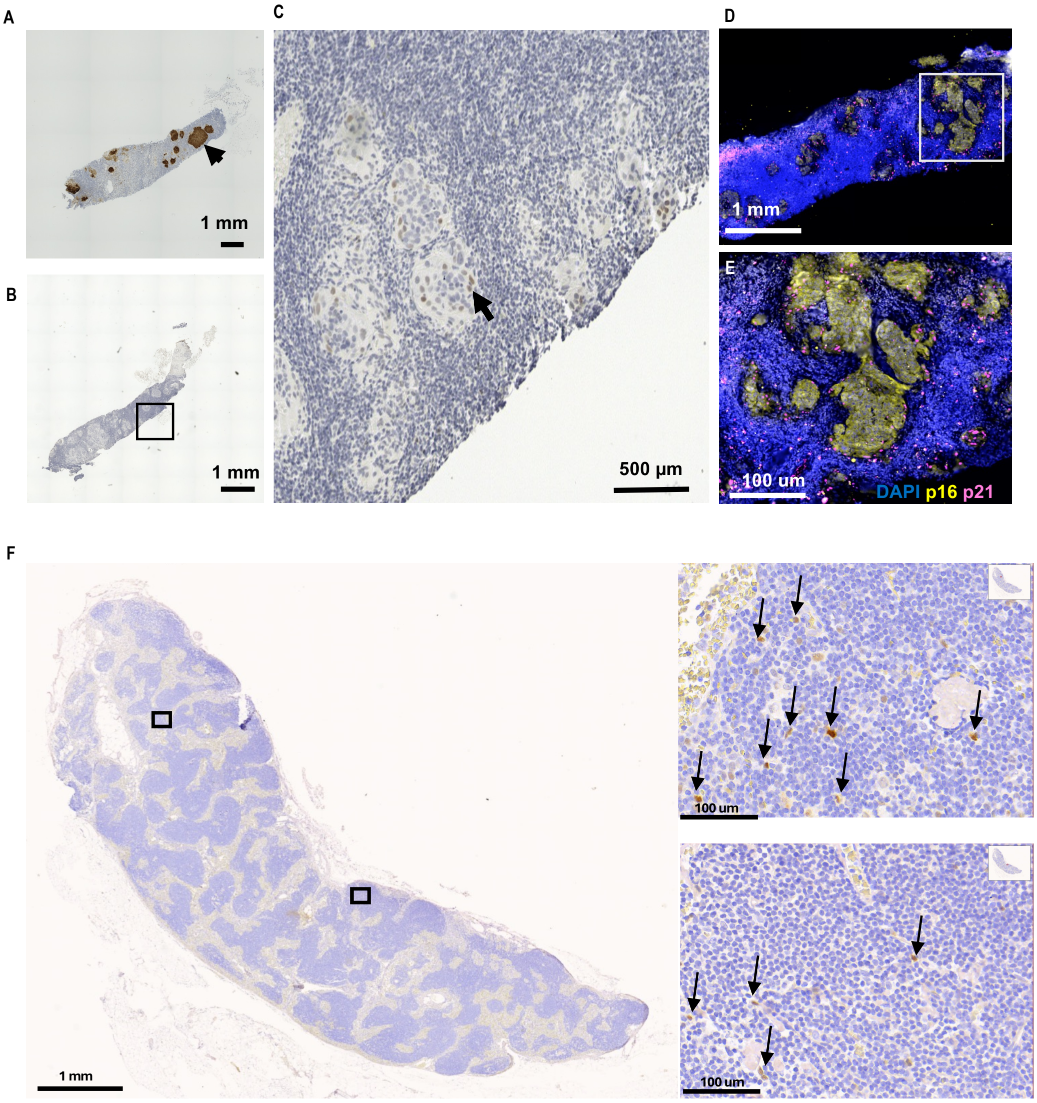

Figure S4. Validation of p16 and p21 antibodies on a p16 positive nasopharyngeal carcinoma and human lymph node samples.
(A-C) Immunohistochemistry (IHC) images showing p16^{+} cells (brown, arrows) within lymph node sections at low (A, B) and higher (C) magnification.
(D-E) Multiplex immunofluorescence images displaying co-localization of p16 (yellow) and p21 (magenta) within tumor regions, counterstained with DAPI (blue).
(F) Representative IHC images of p21 expression across the entire human lymph node section of an old donor-86 years old (left), with magnified insets (right) showing clusters of p21^{+} cells (arrows) localized predominantly in germinal center and perifollicular regions. Scale bars are indicated.

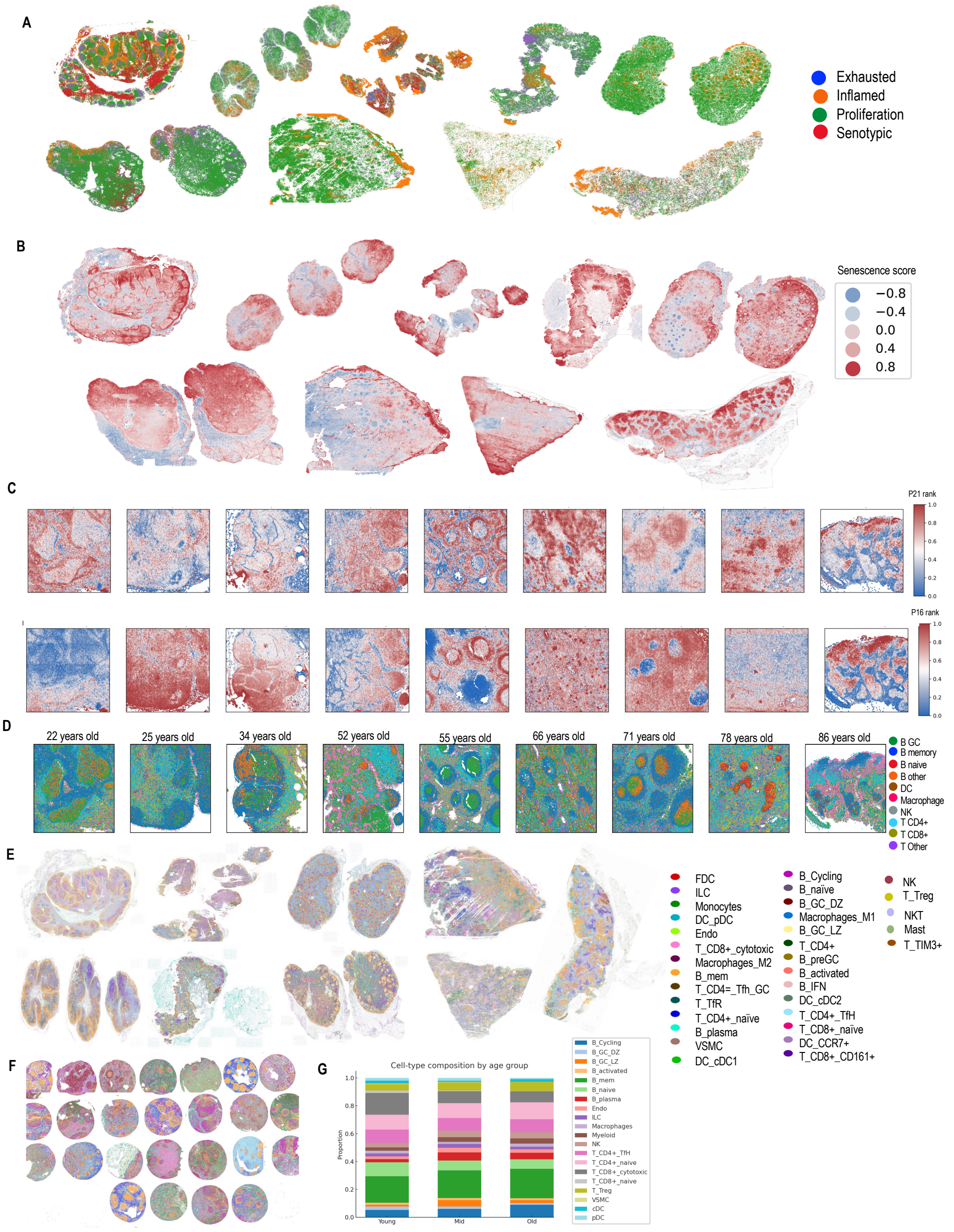

Figure S5. Spatial proteomic mapping of senescence, proliferation, and immune cell composition in human lymph nodes across ages.
(A) Spatial distribution of cellular states (exhausted, inflamed, proliferative, and senescent) within lymph node sections, highlighting heterogeneity across donors.
(B) Spatial proteomic maps senescence score (red) and relative rank across donors, with localized to germinal center regions in 50-70 and >70 years old donors.
(C) Representative high-magnification images illustrating spatial patterns of CDKN2A (p16) and CDKN1A (p21) expression within zoomed in follicles of individual lymph nodes.
(D) CODEX multiplexed immunofluorescence images of lymph node sections from donors aged 22–86 years, displaying cell-type distribution and senescent cell localization.
(E) Integrated cell-type annotation of lymph node sections combining multiplexed protein and scRNA-seq reference data, identifying major immune cell populations.

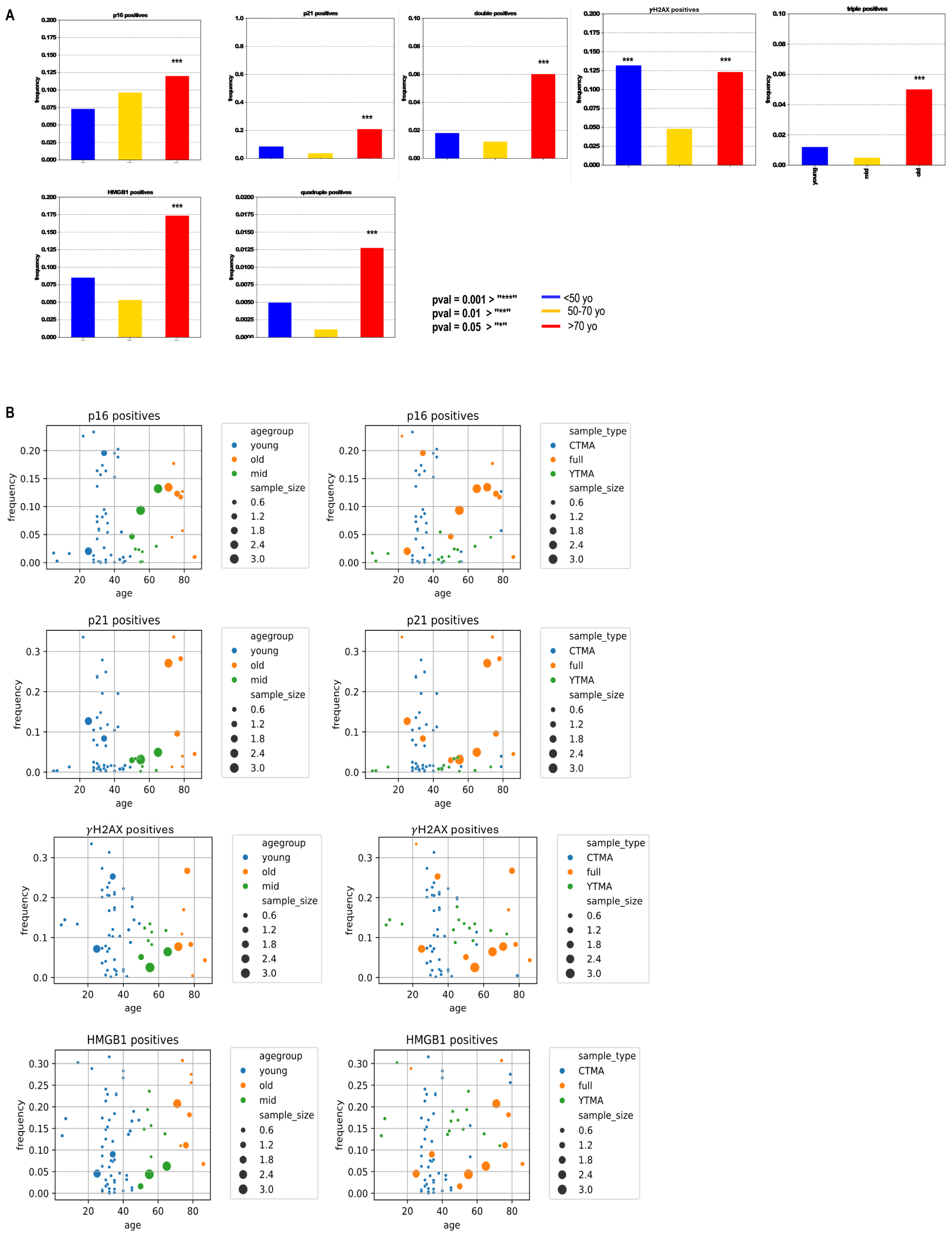

Figure S6. Quantification of senescence marker-positive cells in human lymph nodes across age groups using CODEX.

(A) Bar plots showing the frequency of cells positive for p16, p21, double-positive (p16+p21), γ-H2AX, triple-positive (p16+p21+ γ-H2AX+or HMGB1+), HMGB1, and quadruple (p16+p21+ γ-H2AX+HMGB1+) across three age groups (<50 , 50-70, and >70 years old). Statistical significance is indicated (**p ≤ 0.01; ***p ≤ 0.001).

(B) Scatter plots depicting the frequency of $p16^{+}$, $p21^{+}$, γ-$H2AX^{+}$, and $HMGB1^{+}$ cells as a function of donor age. Data points are color-coded by age group (<50 , 50-70, and >70 years old). and shaped by sample type (CTMA, full, YTMA), with point size indicating sample size (cells in millions).

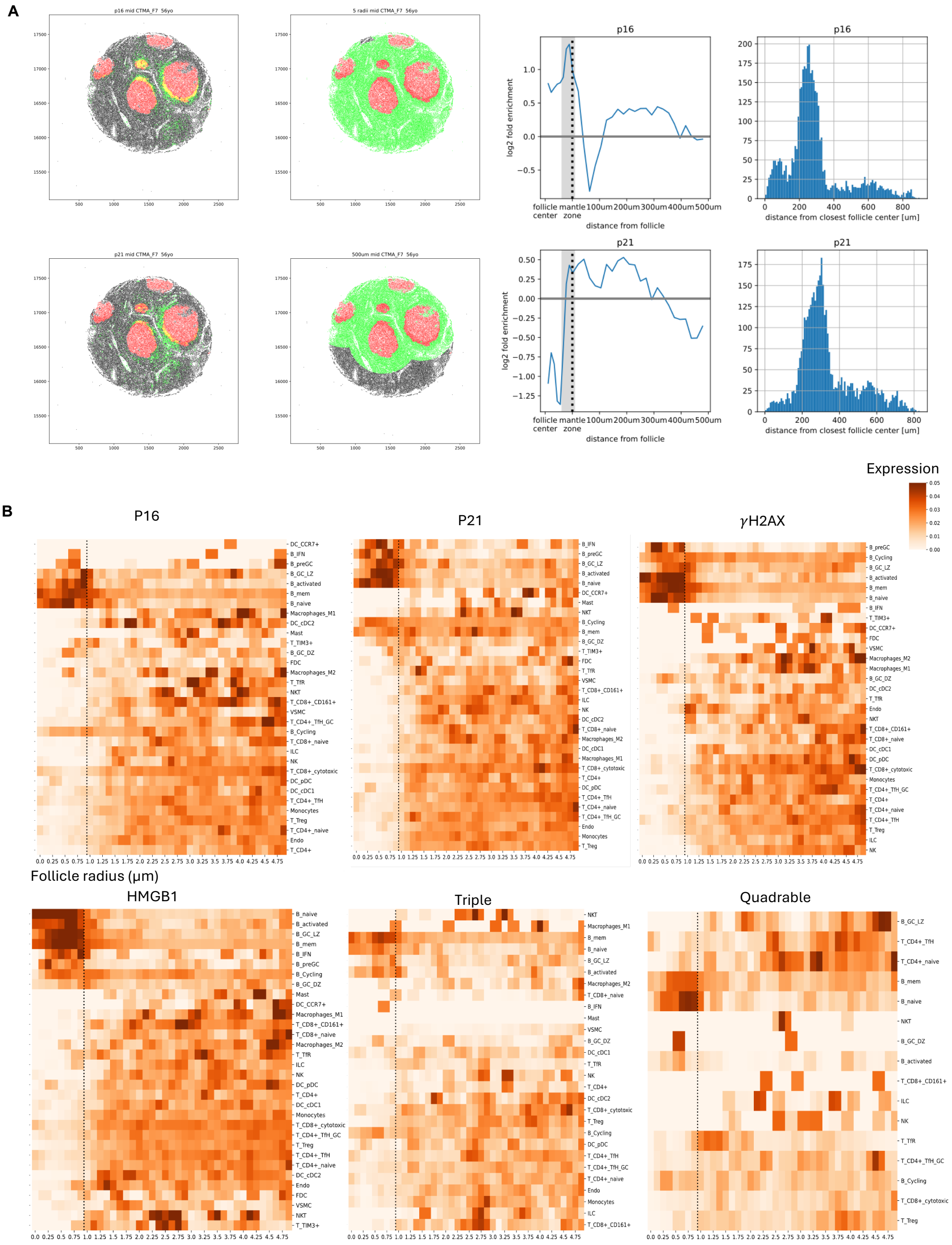

Figure S7. Spatial enrichment of senescence marker-positive cells relative to follicular structures.
(A) Representative images showing follicle identification on a mid-age TMA (56 years old) based on sample-specific CD20 and CD21 expression. For each follicle, a Gaussian mixture model was applied to determine centroid location and morphology. Line plots depict fold-enrichment of p16^{+} and p21^{+} cells relative to distance from follicle centers in both Mahalanobis and Euclidean space. Histograms on the right show the distribution of distances of p16^{+} and p21^{+} cells from the nearest follicle center.
(B) Heatmaps depicting the abundance of cells positive for p16, p21, γ-H2AX, HMGB1, triple-positive, and quadruple-positive markers as a function of follicular radius (x-axis) across various cell types (y-axis).

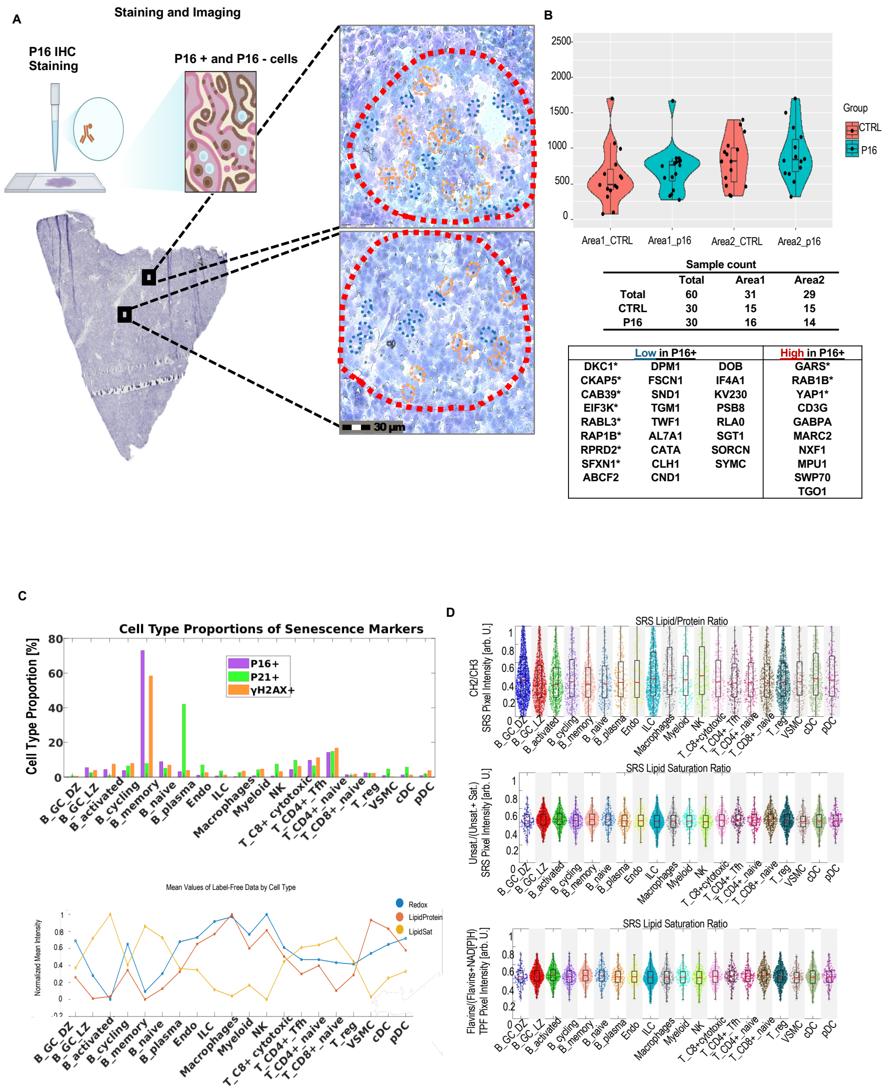

Figure S8. Integrated spatial single-cell proteomics and SRS+CODEX profiling of senescent cells in human lymph nodes.
(A) Human lymph node tissue section from a 78-year-old donor was mounted on a Polyethylene Naphthalate (PEN) membrane for spatial single-cell proteomics. Workflow for isolating single p16^{+} and p16^{-} cells from a fresh frozen human lymph node section (78-year-old donor) mounted on a PEN membrane. Sections were stained with p16-IHC, and target follicles were identified by overlapping p16-IHC images with serial sections stained using a multiplex immune panel (including p16 and p21). Single cells were captured by laser ablation and deposited into nanoPOTS wells, followed by proteomic preparation and LC-MS/MS analysis. Representative images show selected follicles with p16^{+} (orange) and p16^{-} (blue) cells.
(B) Violin plots comparing the median number of quantified protein groups per cell for p16^{+} and p16^{-} cells across two follicular regions. The table lists proteins with significantly different abundance between p16^{+} and p16^{-} cells, as identified by ANOVA, with key proteins enriched or depleted in p16^{+} cells highlighted.
(C) Bar plots showing cell type proportions of p16^{+}, p21^{+}, and γ-H2AX^{+} senescent cells. The line plot below shows mean label-free SRS-derived metabolic features (lipid-to-protein ratio, lipid saturation, and lipid unsaturation) across cell types.
(D) Violin plots displaying SRS-derived lipid/protein ratio, lipid saturation ratio, and lipid unsaturation ratio for major immune cell subsets, overlaid with CODEX-defined cell types.

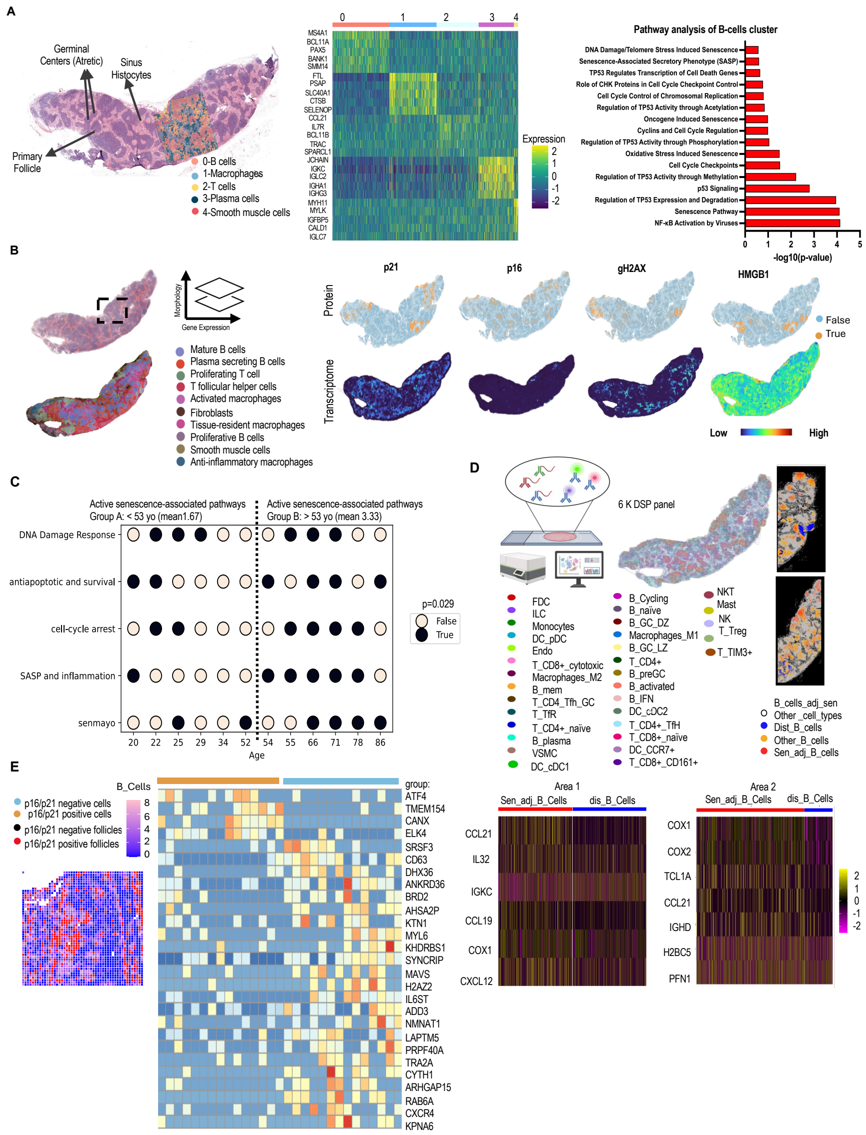

Figure S9. Integration of DBiT-seq, CosMx, and CODEX for spatial and transcriptomic characterization of senescent B cells.
(A) H&E image annotation of lymph node regions (germinal centers, sinuses, primary follicles) overlaid with DBiT-seq transcriptomic profiling. The heatmap shows gene expression in clusters, and pathway analysis highlights pathway enrichment in the B-cells cluster.
(B) Super-resolved transcriptome-wide expression was generated from the DBiT-seq profile using iSTAR and compared with the multiplexed protein profile. Expression patterns of p21, p16, γ-H2AX, and HMGB1 were analyzed across the entire lymph node from an 86-year-old donor.
(C) Dot plot summarizing pathway-level senescence activity across individual donors ordered by age. For each donor, five senescence-related programs (DNA damage response, anti-apoptotic/survival signaling, cell-cycle arrest, SASP/inflammation, and SenMayo) were binarized as active (True, dark) if the pathway score ranked above the cohort median, or inactive (False, light) otherwise.
(D) Integration of CosMx spatial transcriptomics with scRNA-seq reference using MaxFuse, resulting in single-cell level annotation of immune populations. CosMx spatial analysis comparing p16/p21-positive and -negative B cells within follicles. DE gene heatmaps reveal distinct transcriptional profiles of senescent versus non-senescent B cells.
(E) Identification of p16/p21-positive B cells in DBiT-seq regions of interest (ROIs). Heatmaps show differentially expressed genes between p16/p21-positive and -negative pixels within follicular regions.

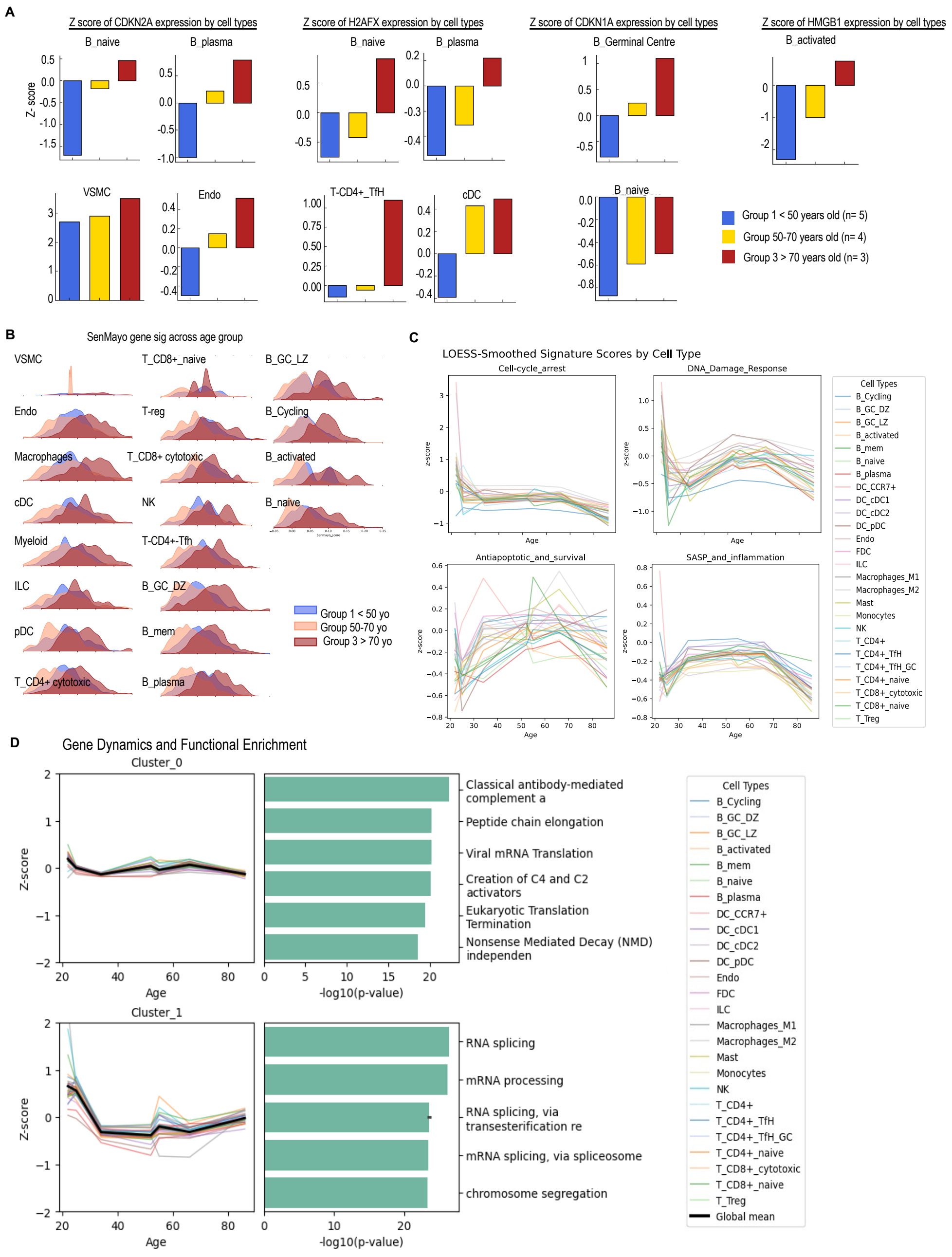

Figure S10. Age-associated transcriptional dynamics and pathway enrichment in lymph node cell types using integrated DBiT-seq and scRNA-seq analysis.

(A) Bar plots showing Z-scores of CDKN2A, H2AFX, CDKN1A, and HMGB1 expression across selected immune and stromal cell types (e.g., B_naive, B_plasma, B_GerminalCentre, VSMC, Endothelial, T_CD4+_TH, cDC) stratified by age group (<50 , 50-70, and >70 years old).

(B) Density plots of SenMayo gene set signature scores across cell types and age groups, highlighting increased senescence-associated transcriptional burden in specific B cell and other immune cell populations with age.

(C) Line plots illustrating age-associated dynamics of DNA damage response, cell cycle arrest, anti-apoptotic/survival, and SASP/inflammation gene programs across cell types.

(D) Gene dynamics and functional enrichment analyses for two identified gene clusters. Cluster_0 is enriched for antibody-mediated complement activation and viral mRNA translation, while Cluster_1 is enriched for RNA splicing, mRNA processing, and chromosome segregation pathways.

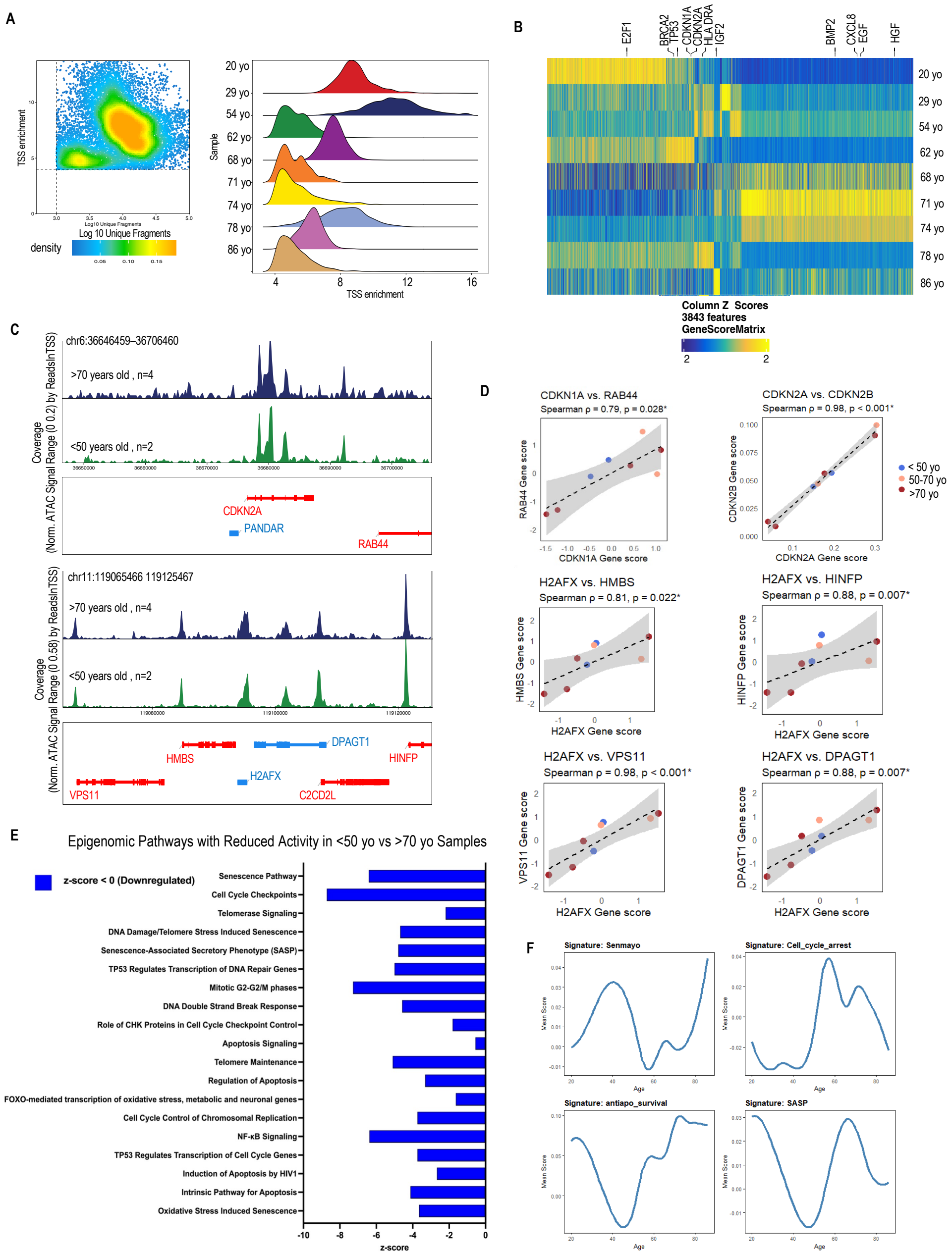

Figure S11. Spatial ATAC-seq analysis reveals age-associated chromatin accessibility changes in senescence-related genes.
(A) Representative density and distribution plots of chromatin accessibility peaks across all cell types identified in spatial ATAC-seq data.
(B) Heatmap of Z-scored chromatin accessibility for senescence-related genes (e.g., CDKN2A, CDKN1A, H2AFX, HMGB1) across donors of different ages.
(C) Genome browser tracks depicting chromatin accessibility peaks at the CDKN2A and H2AFX loci in <50 and >70 years old samples.
(D) Scatter plots showing the correlation between H2AFX or CDKN1A expression and that of neighboring genes (e.g., RAB44, HMBS, HINFP, DPAGT1, VPS11), with Spearman correlation coefficients and associated p-values indicated. Scatter plots showing correlations between H2AFX or CDKN1A gene scores and expression of and associated genes (e.g., HMBS, HINFP, DPAGT1, VPS11, PANDAR, RAB44) gene expression, with Spearman correlation coefficients and p-values indicated.
(E) Bar plot showing pathways that are significantly downregulated in older compared to <50 years old samples based on chromatin accessibility analysis. The x-axis represents the –log10(p-value) of pathway enrichment.
(F) line plots illustrate anti-apoptotic/survival, cell cycle arrest, SASP and SenMayo signature scores across different age.

Supplementary Table 1: Summary of Human Lymph Node Tissue Procurement

Block numbers	Section	Age (yo)	Gender	Location	FF/FFPE	Modality
YHLN-N6	Yale- whole lymph node	63	M	right inguinal	FF	codex / spatial atac / spatial transcript
YHLN-N8	Yale - whole lymph node	29	F	hilar	FF	codex / spatial atac / spatial transcript
YHLN-N9	Yale - whole lymph node	54	F	submental	FF	codex / spatial atac / spatial transcript
YHLN-N17	Yale - whole lymph node	78	F	left axillar	FF	codex / spatial atac / spatial transcript
YHLN-N2	Yale - whole lymph node	62	M	left neck	FF	codex / spatial atac
YHLN-N22	Yale - whole lymph node	22	F	left neck	FFPE	codex
YHLN-N27	Yale - whole lymph node	25	F	right neck	FFPE	codex / spatial transcript
YHLN-N4	Yale - whole lymph node	70	M	right inguinal	FF	-
YHLN-N1	Yale - whole lymph node	73	F	left groin	FF	-
YHLN-N3	Yale - whole lymph node	68	M	left neck	FF	codex / spatial atac
YHLN-N5	Yale - whole lymph node	62	F	left tonsil	FF	-
YHLN-N7	Yale - whole lymph node	75	F	left axillar	FF	-
YHLN-N12	Yale - whole lymph node	20	M	right neck	FF	codex / spatial atac / spatial transcript
YHLN-N13	Yale - whole lymph node	71	F	left inguinal	FF	codex / spatial atac / spatial transcript
YHLN-N14	Yale - whole lymph node	74	M	left neck	FF	codex / spatial atac
YHLN-N15	Yale - whole lymph node	84	F	right axillary	FF	-
YHLN-N16	Yale - whole lymph node	86	M	left neck	FF	codex / spatial atac
YHLN-N18	Yale - whole lymph node	62	M	right neck	FFPE	-
YHLN-N19	Yale - whole lymph node	81	M	right base of tongue	FFPE	-
YHLN-N20	Yale - whole lymph node	66	M	right axillary	FFPE	codex / spatial transcript
YHLN-N21	Yale - whole lymph node	45	F	left axillar	FFPE	-
YHLN-N23	Yale - whole lymph node	52	M	right neck	FFPE	codex / spatial transcript
YHLN-N24	Yale - whole lymph node	74	F	right inguinal	FFPE	-
YHLN-N25	Yale - whole lymph node	74	F	right inguinal	FFPE	-
YHLN-N26	Yale - whole lymph node	86	M	left axillary	FFPE	codex / spatial transcript
YHLN-N28	Yale - whole lymph node	55	M	right neck	FFPE	-

YHLN-N29	Yale - whole lymph node	34	M	left neck	FFPE	codex / spatial transcript
YHLN-N30	Yale - whole lymph node	55	F	left neck	FFPE	codex / spatial transcript
YHLN-N31	Yale - whole lymph node	22	M	right axilla	FFPE	codex
YHLN-N32	Yale - whole lymph node	61	M	right groin	FFPE	-
YHLN-N33	Yale - whole lymph node	63	F	right axilla	FFPE	-
YHLN-N34	Yale-TMA	53	F	left axillary lymph node	FFPE	codex
YHLN-N35	Yale-TMA	65	F	right neck mass	FFPE	codex
YHLN-N36	Yale-TMA	46	M	midline neck	FFPE	codex
YHLN-N37	Yale-TMA	57	F	submental mass	FFPE	codex
YHLN-N38	Yale-TMA	56	F	left level 2 lymph node	FFPE	codex
YHLN-N39	Yale-TMA	17	M	left neck mass	FFPE	codex
YHLN-N40	Yale-TMA	47	F	right neck mass	FFPE	codex
YHLN-N41	Yale-TMA	8	M	left neck mass	FFPE	codex
YHLN-N42	Yale-TMA	45	F	right external jugular lymph	FFPE	codex
YHLN-N43	Yale-TMA	16	M	right sided submental mass	FFPE	codex
YHLN-N44	Yale-TMA	29	F	right neck level 5 lymph node	FFPE	codex
YHLN-N45	Yale-TMA	29	F	submental lymph nodes for lymphoma	FFPE	codex
YHLN-N46	Yale-TMA	32	F	left level 2 lymph node	FFPE	codex
YHLN-N47	Yale-TMA	47	M	right level 2 neck mass	FFPE	codex
YHLN-N48	Yale-TMA	50	F	left groin lymph node 2	FFPE	codex
YHLN-N49	Yale-TMA	6	F	left cervical lymph node	FFPE	codex
YHLN-N50	Yale-TMA	83	M	colon	FFPE	codex
YHLN-N51	Yale-TMA	60	F	right colon	FFPE	codex
YHLN-N52	Yale-TMA	85	M	level 7 lymph node	FFPE	codex
YHLN-N53	Yale-TMA	81	F	right 10 node	FFPE	codex
YHLN-N54	Yale-TMA	58	M	mesenteric lymph node	FFPE	codex
YHLN-N55	Yale-TMA	71	M	pancreas and spleen	FFPE	codex

YHLN-N56	Yale-TMA	68	F	left axilla lymph node	FFPE	codex
N57	Commercial-TMA	30	M	NA	FFPE	codex
N58	Commercial-TMA	34	M	NA	FFPE	codex
N59	Commercial-TMA	30	M	NA	FFPE	codex
N60	Commercial-TMA	40	M	NA	FFPE	codex
N61	Commercial-TMA	32	M	NA	FFPE	codex
N62	Commercial-TMA	35	M	NA	FFPE	codex
N63	Commercial-TMA	28	M	NA	FFPE	codex
N64	Commercial-TMA	38	M	NA	FFPE	codex
N65	Commercial-TMA	30	M	NA	FFPE	codex
N66	Commercial-TMA	35	M	NA	FFPE	codex
N67	Commercial-TMA	36	M	NA	FFPE	codex
N68	Commercial-TMA	31	M	NA	FFPE	codex
N69	Commercial-TMA	28	M	NA	FFPE	codex
N70	Commercial-TMA	31	M	NA	FFPE	codex
N71	Commercial-TMA	33	M	NA	FFPE	codex
N72	Commercial-TMA	40	M	NA	FFPE	codex
N73	Commercial-TMA	28	M	NA	FFPE	codex
N74	Commercial-TMA	32	M	NA	FFPE	codex
N75	Commercial-TMA	32	M	NA	FFPE	codex
N76	Commercial-TMA	45	M	NA	FFPE	codex
N77	Commercial-TMA	35	M	NA	FFPE	codex
N78	Commercial-TMA	79	F	NA	FFPE	codex
N79	Commercial-TMA	42	M	NA	FFPE	codex
N80	Commercial-TMA	56	M	NA	FFPE	codex

Supplementary Table 2: CODEX Antibody panel

Antibody	Antibody_RRID	Antibody source	Dilution_factor	Reporter_Cat#	Reporter source
Bcl-2	AB_2936078	Akoya	200	4550089	Akoya
Beta-actin	AB_3478101	Akoya	200	4450040	Akoya
CD107a	AB_3474468	Akoya	200	4550098	Akoya
CD11c	AB_3083459	Akoya	200	4550114	Akoya
CD14	AB_3083457	Akoya	400	4450047	Akoya
CD141	AB_3082975	Akoya	200	4250097	Akoya
CD163	AB_2935895	Akoya	200	4250079	Akoya
CD20	AB_3094498	Akoya	200	4450094	Akoya
CD21	AB_3474900	Akoya	400	4450027	Akoya
CD31	AB_2915935	Akoya	100	4450017	Akoya
CD34	AB_2909512	Akoya	100	4250057	Akoya
CD38	AB_3082976	Akoya	200	4250080	Akoya
CD39	AB_3096410	Akoya	200	4250076	Akoya
CD3e	AB_2936080	Akoya	200	4550119	Akoya
CD4	AB_3094499	Akoya	200	4550112	Akoya
CD44	AB_2936081	Akoya	400	4450041	Akoya
CD45	AB_2915946	Akoya	400	4450042	Akoya
CD45RO	AB_2895053	Akoya	200	4250023	Akoya
CD66	AB_3475664	Akoya	200	4550001	Akoya
CD68	AB_2935894	Akoya	400	4550113	Akoya
CD8	AB_2915960	Akoya	200	4250012	Akoya
CDKN2A/ p16	AB_2078303	Thermo Fisher Scientific	75	10883-1-AP	Akoya
p21	AB_3099451	Cell Signaling Technology	200	19399	Akoya
CXCL13	AB_2746222	Abcam	400	38738A03	Akoya
CXCR5	AB_394324	BD Biosciences	200	552032	Akoya
Collagen IV	AB_2927676	Akoya	200	4550122	Akoya
E-cadherin	AB_2895057	Akoya	200	4250021	Akoya
EpCAM	AB_2935888	Akoya	200	4550088	Akoya
FOXP3	AB_2927679	Akoya	200	4550071	Akoya
Granzyme B	AB_3472025	Akoya	200	4250055	Akoya
HLA-A	AB_3094501	Akoya	200	4250100	Akoya
HLA-DR	AB_3080864	Akoya	200	4550118	Akoya
HLA-E	AB_3478569	Akoya	200	4250065	Akoya
HMGB1	AB_2049739	Thermo Fisher Scientific	150	6550032	Akoya
ICOS	AB_3096408	Akoya	200	4550117	Akoya

IDO1	AB_3476035	Akoya	200	4550123	Akoya
IFN-G	AB_3476455	Akoya	200	4250062	Akoya
Ki67	AB_3094497	Akoya	200	4450096	Akoya
LAG3	AB_3096409	Akoya	100	4550058	Akoya
LMP1	AB_1566182	Akoya	50	ab78113	Akoya
MPO	AB_2927678	Akoya	200	4250083	Akoya
Mac2/Galectin-3	AB_3477119	Akoya	200	4450034	Akoya
PCNA	AB_2936083	Akoya	200	4550124	Akoya
PD-L1	AB_3096406	Akoya	100	4550072	Akoya
PD1	AB_3096407	Akoya	200	4550038	Akoya
Pan-Cytokeratin	AB_3083456	Akoya	200	4450020	Akoya
Podoplanin	AB_3082979	Akoya	200	4250094	Akoya
SMA	AB_2936084	Akoya	200	4450049	Akoya
TOX	AB_3477620	Akoya	200	4250067	Akoya
VISTA	AB_3479195	Akoya	150	4250063	Akoya
Vimentin	AB_2935889	Akoya	200	4450050	Akoya
yH2AX	AB_315795	Akoya	300	613402	Akoya

Supplementary Table 3: DNA oligos for PCR, ligation and library preparation- spatial transcriptome

Oligo	Sequence
RT primer	/5Phos/CATCGGCGTACGACTNNNNNNNNNN/iBiodT/TTTTTTTTTTTTTTTVN
Ligation linker 1	AGTCGTACGCCGATGCGAAACATCGGCCAC
Ligation linker 2	CGAATGCTCTGGCCTCTCAAGCACGTGGAT
PCR Primer 1	CAAGCGTTGGCTTCTCGCATCT
PCR Primer 2	AAGCAGTGGTATCAACGCAGAGT
Template Switch Oligo	AAGCAGTGGTATCAACGCAGAGTGAATrGrG+G
dN-SMRT	AAGCAGTGGTATCAACGCAGAGTGANNNGGNNNB
P5 oligo for FFPE direct ligation	AATGATACGGCGACCACCGAGATCTACACTAGATCGCTCGTCGGCAGCGTCAGATGTGTATAAGAGACAGAAGCAGTGGTATCAACGCAGAGT
N501	AATGATACGGCGACCACCGAGATCTACACTAGATCGCTCGTCGGCAGCGTCAGATGTGTATAAGAGACAG
N701	CAAGCAGAAGACGGCATACGAGATTCGCCTTAGTCTCGTGGGCTCGGAGATGTGTATAAGAGACAGCAAGCGTTGGCTTCTCGCATCT
N702	CAAGCAGAAGACGGCATACGAGATCTAGTACGGTCTCGTGGGCTCGGAGATGTGTATAAGAGACAGCAAGCGTTGGCTTCTCGCATCT
N703	CAAGCAGAAGACGGCATACGAGATTTCTGCCTGTCTCGTGGGCTCGGAGATGTGTATAAGAGACAGCAAGCGTTGGCTTCTCGCATCT
N704	CAAGCAGAAGACGGCATACGAGATGCTCAGGAGTCTCGTGGGCTCGGAGATGTGTATAAGAGACAGCAAGCGTTGGCTTCTCGCATCT
N705	CAAGCAGAAGACGGCATACGAGATAGGAGTCCGTCTCGTGGGCTCGGAGATGTGTATAAGAGACAGCAAGCGTTGGCTTCTCGCATCT
N706	CAAGCAGAAGACGGCATACGAGATCATGCCTAGTCTCGTGGGCTCGGAGATGTGTATAAGAGACAGCAAGCGTTGGCTTCTCGCATCT
N707	CAAGCAGAAGACGGCATACGAGATGTAGAGAGGTCTCGTGGGCTCGGAGATGTGTATAAGAGACAGCAAGCGTTGGCTTCTCGCATCT

Supplementary Table 4: DNA barcode B sequences- spatial ATAC-seq

Oligo	Sequence
Tn5MErev	/5Phos/CTGTCTCTTATACACATCT
Tn5ME-A	/5Phos/TCGTCGGCAGCGTCAGATGTGTATAAGAGACAG
Tn5ME-B	GTCTCGTGGGCTCGGAGATGTGTATAAGAGACAG
Ligation linker 1	AGTCGTACGCCGATGCTGTCTCTTATACACATCT
Ligation linker 2	CGAATGCTCTGGCCTCTCAAGCACGTGGAT
Custom Read 1 Primer	GCCTGTCCGCGGAAGCAGTGGTATCAACGCAGAGTAC
N501	AATGATACGGCGACCACCGAGATCTACACTAGATCGCTCGTCGGCAG CGTCAGATGTGTATAAGAGACAG
N701	CAAGCAGAAGACGGCATACGAGATTCGCCTTAGTCTCGTGGGCTCG G AGATGTGTATAAGAGACAGCAAGCGTTGGCTTCTCGCATCT
N702	CAAGCAGAAGACGGCATACGAGATCTAGTACGGTCTCGTGGGCTCG G AGATGTGTATAAGAGACAGCAAGCGTTGGCTTCTCGCATCT
N703	CAAGCAGAAGACGGCATACGAGATTTCTGCCTGTCTCGTGGGCTCGG AGATGTGTATAAGAGACAGCAAGCGTTGGCTTCTCGCATCT
N704	CAAGCAGAAGACGGCATACGAGATGCTCAGGAGTCTCGTGGGCTCG G AGATGTGTATAAGAGACAGCAAGCGTTGGCTTCTCGCATCT
N705	CAAGCAGAAGACGGCATACGAGATAGGAGTCCGTCTCGTGGGCTCG G AGATGTGTATAAGAGACAGCAAGCGTTGGCTTCTCGCATCT

Supplementary Table 5: DNA barcode A sequences

Barcode A	Sequence
Barcode A-1	/5Phos/AGGCCAGAGCATTCGAACGTGATGTGGCCGATGTTTCG
Barcode A-2	/5Phos/AGGCCAGAGCATTCGAAACATCGGTGGCCGATGTTTCG
Barcode A-3	/5Phos/AGGCCAGAGCATTCGATGCCTAAGTGGCCGATGTTTCG
Barcode A-4	/5Phos/AGGCCAGAGCATTCGAGTGGTCAGTGGCCGATGTTTCG
Barcode A-5	/5Phos/AGGCCAGAGCATTCGACCACTGTGTGGCCGATGTTTCG
Barcode A-6	/5Phos/AGGCCAGAGCATTCGACATTGGCGTGGCCGATGTTTCG
Barcode A-7	/5Phos/AGGCCAGAGCATTCGCAGATCTGGTGGCCGATGTTTCG
Barcode A-8	/5Phos/AGGCCAGAGCATTCGCATCAAGTGTGGCCGATGTTTCG
Barcode A-9	/5Phos/AGGCCAGAGCATTCGCGCTGATCGTGGCCGATGTTTCG
Barcode A-10	/5Phos/AGGCCAGAGCATTCGACAAGCTAGTGGCCGATGTTTCG
Barcode A-11	/5Phos/AGGCCAGAGCATTCGCTGTAGCCGTGGCCGATGTTTCG
Barcode A-12	/5Phos/AGGCCAGAGCATTCGAGTACAAGGTGGCCGATGTTTCG
Barcode A-13	/5Phos/AGGCCAGAGCATTCGAACAACCAGTGGCCGATGTTTCG
Barcode A-14	/5Phos/AGGCCAGAGCATTCGAACCGAGAGTGGCCGATGTTTCG
Barcode A-15	/5Phos/AGGCCAGAGCATTCGAACGCTTAGTGGCCGATGTTTCG
Barcode A-16	/5Phos/AGGCCAGAGCATTCGAAGACGGAGTGGCCGATGTTTCG
Barcode A-17	/5Phos/AGGCCAGAGCATTCGAAGGTACAGTGGCCGATGTTTCG
Barcode A-18	/5Phos/AGGCCAGAGCATTCGACACAGAAGTGGCCGATGTTTCG
Barcode A-19	/5Phos/AGGCCAGAGCATTCGACAGCAGAGTGGCCGATGTTTCG
Barcode A-20	/5Phos/AGGCCAGAGCATTCGACCTCCAAGTGGCCGATGTTTCG
Barcode A-21	/5Phos/AGGCCAGAGCATTCGACGCTCGAGTGGCCGATGTTTCG
Barcode A-22	/5Phos/AGGCCAGAGCATTCGACGTATCAGTGGCCGATGTTTCG
Barcode A-23	/5Phos/AGGCCAGAGCATTCGACTATGCAGTGGCCGATGTTTCG
Barcode A-24	/5Phos/AGGCCAGAGCATTCGAGAGTCAAGTGGCCGATGTTTCG
Barcode A-25	/5Phos/AGGCCAGAGCATTCGAGATCGCAGTGGCCGATGTTTCG
Barcode A-26	/5Phos/AGGCCAGAGCATTCGAGCAGGAAGTGGCCGATGTTTCG
Barcode A-27	/5Phos/AGGCCAGAGCATTCGAGTCACTAGTGGCCGATGTTTCG
Barcode A-28	/5Phos/AGGCCAGAGCATTCGATCCTGTAGTGGCCGATGTTTCG
Barcode A-29	/5Phos/AGGCCAGAGCATTCGATTGAGGAGTGGCCGATGTTTCG
Barcode A-30	/5Phos/AGGCCAGAGCATTCGCAACCACAGTGGCCGATGTTTCG
Barcode A-31	/5Phos/AGGCCAGAGCATTCGGACTAGTAGTGGCCGATGTTTCG
Barcode A-32	/5Phos/AGGCCAGAGCATTCGCAATGGAAGTGGCCGATGTTTCG
Barcode A-33	/5Phos/AGGCCAGAGCATTCGCACTTCGAGTGGCCGATGTTTCG
Barcode A-34	/5Phos/AGGCCAGAGCATTCGCAGCGTTAGTGGCCGATGTTTCG
Barcode A-35	/5Phos/AGGCCAGAGCATTCGCATACCAAGTGGCCGATGTTTCG
Barcode A-36	/5Phos/AGGCCAGAGCATTCGCCAGTTCAGTGGCCGATGTTTCG
Barcode A-37	/5Phos/AGGCCAGAGCATTCGCCGAAGTAGTGGCCGATGTTTCG
Barcode A-38	/5Phos/AGGCCAGAGCATTCGCCGTGAGAGTGGCCGATGTTTCG
Barcode A-39	/5Phos/AGGCCAGAGCATTCGCCTCCTGAGTGGCCGATGTTTCG
Barcode A-40	/5Phos/AGGCCAGAGCATTCGCGAACTTAGTGGCCGATGTTTCG
Barcode A-41	/5Phos/AGGCCAGAGCATTCGCGACTGGAGTGGCCGATGTTTCG

Barcode A-42	/5Phos/AGGCCAGAGCATTCGCGCATACAGTGGCCGATGTTTCG
Barcode A-43	/5Phos/AGGCCAGAGCATTCGCTCAATGAGTGGCCGATGTTTCG
Barcode A-44	/5Phos/AGGCCAGAGCATTCGCTGAGCCAGTGGCCGATGTTTCG
Barcode A-45	/5Phos/AGGCCAGAGCATTCGCTGGCATAGTGGCCGATGTTTCG
Barcode A-46	/5Phos/AGGCCAGAGCATTCGGAATCTGAGTGGCCGATGTTTCG
Barcode A-47	/5Phos/AGGCCAGAGCATTCGCAAGACTAGTGGCCGATGTTTCG
Barcode A-48	/5Phos/AGGCCAGAGCATTCGGAGCTGAAGTGGCCGATGTTTCG
Barcode A-49	/5Phos/AGGCCAGAGCATTCGGATAGACAGTGGCCGATGTTTCG
Barcode A-50	/5Phos/AGGCCAGAGCATTCGGCCACATAGTGGCCGATGTTTCG

Supplementary Table 6: DNA barcode B sequences

Barcode B	Sequence
Barcode B-1	CAAGCGTTGGCTTCTCGCATCTAACGTGATATCCACGTGCTTGAG
Barcode B-2	CAAGCGTTGGCTTCTCGCATCTAAACATCGATCCACGTGCTTGAG
Barcode B-3	CAAGCGTTGGCTTCTCGCATCTATGCCTAAATCCACGTGCTTGAG
Barcode B-4	CAAGCGTTGGCTTCTCGCATCTAGTGGTCAATCCACGTGCTTGAG
Barcode B-5	CAAGCGTTGGCTTCTCGCATCTACCACTGTATCCACGTGCTTGAG
Barcode B-6	CAAGCGTTGGCTTCTCGCATCTACATTGGCATCCACGTGCTTGAG
Barcode B-7	CAAGCGTTGGCTTCTCGCATCTCAGATCTGATCCACGTGCTTGAG
Barcode B-8	CAAGCGTTGGCTTCTCGCATCTCATCAAGTATCCACGTGCTTGAG
Barcode B-9	CAAGCGTTGGCTTCTCGCATCTCGCTGATCATCCACGTGCTTGAG
Barcode B-10	CAAGCGTTGGCTTCTCGCATCTACAAGCTAATCCACGTGCTTGAG
Barcode B-11	CAAGCGTTGGCTTCTCGCATCTCTGTAGCCATCCACGTGCTTGAG
Barcode B-12	CAAGCGTTGGCTTCTCGCATCTAGTACAAGATCCACGTGCTTGAG
Barcode B-13	CAAGCGTTGGCTTCTCGCATCTAACAACCAATCCACGTGCTTGAG
Barcode B-14	CAAGCGTTGGCTTCTCGCATCTAACCGAGAATCCACGTGCTTGAG
Barcode B-15	CAAGCGTTGGCTTCTCGCATCTAACGCTTAATCCACGTGCTTGAG
Barcode B-16	CAAGCGTTGGCTTCTCGCATCTAAGACGGAATCCACGTGCTTGAG
Barcode B-17	CAAGCGTTGGCTTCTCGCATCTAAGGTACAATCCACGTGCTTGAG
Barcode B-18	CAAGCGTTGGCTTCTCGCATCTACACAGAAATCCACGTGCTTGAG
Barcode B-19	CAAGCGTTGGCTTCTCGCATCTACAGCAGAATCCACGTGCTTGAG
Barcode B-20	CAAGCGTTGGCTTCTCGCATCTACCTCCAAATCCACGTGCTTGAG
Barcode B-21	CAAGCGTTGGCTTCTCGCATCTACGCTCGAATCCACGTGCTTGAG
Barcode B-22	CAAGCGTTGGCTTCTCGCATCTACGTATCAATCCACGTGCTTGAG
Barcode B-23	CAAGCGTTGGCTTCTCGCATCTACTATGCAATCCACGTGCTTGAG
Barcode B-24	CAAGCGTTGGCTTCTCGCATCTAGAGTCAAATCCACGTGCTTGAG
Barcode B-25	CAAGCGTTGGCTTCTCGCATCTAGATCGCAATCCACGTGCTTGAG
Barcode B-26	CAAGCGTTGGCTTCTCGCATCTAGCAGGAAATCCACGTGCTTGAG
Barcode B-27	CAAGCGTTGGCTTCTCGCATCTAGTCACTAATCCACGTGCTTGAG
Barcode B-28	CAAGCGTTGGCTTCTCGCATCTATCCTGTAATCCACGTGCTTGAG
Barcode B-29	CAAGCGTTGGCTTCTCGCATCTATTGAGGAATCCACGTGCTTGAG
Barcode B-30	CAAGCGTTGGCTTCTCGCATCTCAACCACAATCCACGTGCTTGAG
Barcode B-31	CAAGCGTTGGCTTCTCGCATCTGACTAGTAATCCACGTGCTTGAG
Barcode B-32	CAAGCGTTGGCTTCTCGCATCTCAATGGAAATCCACGTGCTTGAG
Barcode B-33	CAAGCGTTGGCTTCTCGCATCTCACTTCGAATCCACGTGCTTGAG
Barcode B-34	CAAGCGTTGGCTTCTCGCATCTCAGCGTTAATCCACGTGCTTGAG
Barcode B-35	CAAGCGTTGGCTTCTCGCATCTCATACCAAATCCACGTGCTTGAG
Barcode B-36	CAAGCGTTGGCTTCTCGCATCTCCAGTTCAATCCACGTGCTTGAG
Barcode B-37	CAAGCGTTGGCTTCTCGCATCTCCGAAGTAATCCACGTGCTTGAG
Barcode B-38	CAAGCGTTGGCTTCTCGCATCTCCGTGAGAATCCACGTGCTTGAG
Barcode B-39	CAAGCGTTGGCTTCTCGCATCTCCTCCTGAATCCACGTGCTTGAG
Barcode B-40	CAAGCGTTGGCTTCTCGCATCTCGAACTTAATCCACGTGCTTGAG
Barcode B-41	CAAGCGTTGGCTTCTCGCATCTCGACTGGAATCCACGTGCTTGAG

Barcode B-42	CAAGCGTTGGCTTCTCGCATCTCGCATACAATCCACGTGCTTGAG
Barcode B-43	CAAGCGTTGGCTTCTCGCATCTCTCAATGAATCCACGTGCTTGAG
Barcode B-44	CAAGCGTTGGCTTCTCGCATCTCTGAGCCAATCCACGTGCTTGAG
Barcode B-45	CAAGCGTTGGCTTCTCGCATCTCTGGCATAATCCACGTGCTTGAG
Barcode B-46	CAAGCGTTGGCTTCTCGCATCTGAATCTGAATCCACGTGCTTGAG
Barcode B-47	CAAGCGTTGGCTTCTCGCATCTCAAGACTAATCCACGTGCTTGAG
Barcode B-48	CAAGCGTTGGCTTCTCGCATCTGAGCTGAAATCCACGTGCTTGAG
Barcode B-49	CAAGCGTTGGCTTCTCGCATCTGATAGACAATCCACGTGCTTGAG
Barcode B-50	CAAGCGTTGGCTTCTCGCATCTGCCACATAATCCACGTGCTTGAG

Supplementary Table 7: Concordant and discordant features of senescence and aging programs across multi-modal spatial and single-cell datasets

Function	scRNA-seq	Spatial Proteomics	Proteometabolic of GC	Spatial Transcriptome	Spatial Epigenome (ATAC/motifs)	Concordant vs discordant between modalities
Age inflection	Young to mid-age modest; ≥70 strong shift (PBMC separation; LN programs increase late)	Hotspots emerge/enrich in older follicles/GC	Older p16⁺ niche shows stronger molecular remodeling	≥70 shows higher SenMayo, cell-cycle arrest, DDR, SASP/inflammation (global trend)	Old vs young shows broad accessibility shifts at senescence/stress loci	Concordant: multiple layers support late-life acceleration (~70)
Senescence signature	SenMayo gene set in older donors; PBMC enriched in CD4 T, CD8 T, NK; LN enriched in B_naive, B_GC_LZ/DZ, B_plasma, B_cycling	p16/p21/HMGB1/ γ-H2AX increase; shifts from diffuse to GC-centered hotspots	Senescent follicular cells show lipid saturation + oxidized state	Senescence markers sparse at transcriptome level for p16, stronger at protein; p21 and HMGB1 aligns better; programs enriched across follicles/cortex/interfollicular	Accessibility gains at senescence/stress programs; motif programs consistent with stress/inflammation	Concordant: senescence increases with age; discordant nuance: p16 stronger at protein than transcriptome
Cell-cycle arrest	CDKN2A, CDKN1A, TP53, RB1 enriched in older subsets	p16+p21 co-expression increases with age	Suppressed protein production/translation in GC niche aligns with arrest	p16/p21-positive niches enriched for cell-cycle checkpoint/stress programs	Chromatin opening at/near CDKN1A/2A; TF programs support arrest/stress	Concordant: arrest appears at RNA/protein/chromatin levels
DDR / genomic stress	DDR transcripts modest attenuation overall (H2AFX, TP53BP1, TRF2, SUV39H1/2, EZH2, LMNB1, HMGA1/2, LINE-1); retained increases suggest compensation	p16/p21 hotspots in older follicles	Telomere/maintenance stress (DKC1); stress proteins (RAB1B, MARC2); mitochondrial shift (SFX1)	Older regions show DDR enrichment; senescent B cells show stress programs	H2AFX accessibility; stress/senescence pathway accessibility	Partially discordant: DDR transcripts may dampen while γ-H2AX/telomere-proteostasis stress increases
Inflammaging / SASP	PBMC inflammatory/SASP increases with age; senescent B-cell SASP	HMGB1 strong; follicular remodeling with senescenc	Inflammatory/stress rewiring; CXCR4 signaling changes	Older samples show immune remodeling; senescent B niches show inflammatory	Motifs enriched for inflammatory/stress TFs: AP-1 (FOS/JUN);	Concordant: inflammatory/stress programs rise with age

	genes (CD63, MAVS, IL6ST, CXCR4, CYTH1, KTN1)	e marker enrichment		pathway enrichment	NF-κB programs	
B-cell–centric LN aging	LN senescence enriched in B cells; CD22–PTPRC (CD45) inhibitory axis; SEMA4 pathway; TBX21 (T-bet) ABCs; CDKN2A/H2AFX in B_cycling/B_GC_DZ	Strongest age-linked increases in follicular B compartments; GC_“SenSpots”	B-memory primary reservoir (~75% p16^{+}; ~60% γ-H2AX^{+}); lipid/oxidative shift	Follicular B-cell niches show strong senescence pathway enrichment; p16/p21 double^{+} B cells show NF-κB/p53/checkpoint programs	B subsets show strongest accessibility changes; senescence/SASP loci open; tissue remodeling pathways	Highly concordant: LN senescence is B-cell + follicle/GC centered across modalities
Immune remodeling genes and motifs	Age-associated upregulation of antigen-presentation and inflammatory genes across LN immune subsets (B cells, macrophages, cDCs; e.g., CXCL8, BMP2)	CODEX shows increased HMGB1, p16, p21, /γ-H2AX in follicular/APC-rich regions	Senescent GC niches show metabolic and stress rewiring (lipid accumulation, oxidative, ER/mitochondrial stress)	Older donors enriched: HLA-A/B/C, HLA-DRA, HLA-DMB, FCGR2A, HES7; younger: GPR62, TSPYL10, THSD8, LBX1	Motifs: ZKSCAN5, FOS::JUN, ATF6, TCFL5; old gains: KLF1/3/5/6, NFY, SP1/SP2, HOXA9; young: MEF2A, NR2F2	Concordant: HLA/FCGR2A reflects antigen presentation/myeloid activation; AP-1/KLF/ NF-κB support inflammation/senescence
Late-age reprogramming modules	Clusters 2–3: metabolic adaptation + late-life inflammation/remodeling (neutrophil degranulation, ferroptosis, motility, wound response)	Hotspots intensify in late age	Metabolic shift supports lipid/oxidative stress	Late-life regions show stronger senescence/inflammation programs	Accessibility/pathways increase for stress-response programs	Concordant: late-life shift couples metabolism + remodeling/inflammation
